# Bis(3-methyl­pyridinium) tetra­chlorido­cuprate(II)

**DOI:** 10.1107/S1600536809007818

**Published:** 2009-03-11

**Authors:** Nallathambi Sengottvelan, You-Soon Lee, Hyun-Soo Lim, Young-Inn Kim, Sung Kwon Kang

**Affiliations:** a Department of Chemistry Education and Center for Plastic Information Systems, Pusan National University, Pusan 609-735, Republic of Korea; b Department of Chemistry, Chungnam National University, Daejeon 305-764, Republic of Korea

## Abstract

The title compound, (C_6_H_8_N)_2_[CuCl_4_], is composed of two 3-methyl­pyridinium cation and one tetra­chloridocuprate(II) anion. The geometry around the copper(II) ion is that of a distorted tetra­hedron. In the crystal structure, the anions and cations are linked by three different N—H⋯Cl hydrogen bonds. In addition, the crystal structure exhibits aromatic π–π inter­actions between the pyridinium rings of two discrete units [centroid–centroid distance = 3.704 (2) Å].

## Related literature

For general background on the influence of crystal-packing forces on the geometry of the tetrahalogenidocuprate(II) species, see: Schneider *et al.* (2007 ▶); Parent *et al.* (2007 ▶); Haddad *et al.* (2006 ▶); Marzotto *et al.* (2001 ▶); Choi *et al.* (2002 ▶); Awwadi *et al.* (2007 ▶). For the electronic spectrum in DMF solution, see Lee *et al.* (2002 ▶). For related literature, see: Lee *et al.* (2008 ▶).
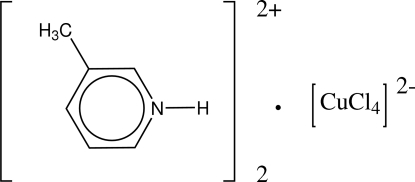


## Experimental

### 

#### Crystal data

(C_6_H_8_N)_2_[CuCl_4_]
*M* *_r_* = 393.61
Monoclinic, 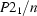

*a* = 9.0438 (3) Å
*b* = 13.0530 (4) Å
*c* = 13.7391 (5) Å
β = 103.541 (2)°
*V* = 1576.80 (9) Å^3^
*Z* = 4
Mo *K*α radiation
μ = 2.05 mm^−1^
*T* = 123 K
0.25 × 0.24 × 0.23 mm

#### Data collection

Bruker SMART CCD area-detector diffractometer
Absorption correction: multi-scan (*SADABS*; Bruker, 2002 ▶) *T*_min_ = 0.603, *T*_max_ = 0.62
16009 measured reflections
3899 independent reflections
3429 reflections with *I* > 2σ(*I*)
*R*_int_ = 0.036

#### Refinement

*R*[*F* ^2^ > 2σ(*F* ^2^)] = 0.024
*wR*(*F* ^2^) = 0.062
*S* = 1.04
3899 reflections
182 parameters
H atoms treated by a mixture of independent and constrained refinement
Δρ_max_ = 0.36 e Å^−3^
Δρ_min_ = −0.35 e Å^−3^

### 

Data collection: *SMART* (Bruker, 2002 ▶); cell refinement: *SAINT* (Bruker, 2002 ▶); data reduction: *SAINT*; program(s) used to solve structure: *SHELXS97* (Sheldrick, 2008 ▶); program(s) used to refine structure: *SHELXL97* (Sheldrick, 2008 ▶); molecular graphics: *ORTEP-3 for Windows* (Farrugia, 1997 ▶) and *DIAMOND* (Brandenburg, 1998 ▶); software used to prepare material for publication: *WinGX* (Farrugia, 1999 ▶).

## Supplementary Material

Crystal structure: contains datablocks global, I. DOI: 10.1107/S1600536809007818/lx2094sup1.cif

Structure factors: contains datablocks I. DOI: 10.1107/S1600536809007818/lx2094Isup2.hkl

Additional supplementary materials:  crystallographic information; 3D view; checkCIF report

## Figures and Tables

**Table 1:** Hydrogen-bond geometry (Å, °)

*D*—H⋯*A*	*D*—H	H⋯*A*	*D*⋯*A*	*D*—H⋯*A*
N1—H1⋯Cl1	0.80 (2)	2.44 (2)	3.136 (2)	146 (2)
N2—H2⋯Cl1	0.81 (2)	2.66 (2)	3.270 (2)	134 (2)
N2—H2⋯Cl2	0.81 (2)	2.50 (2)	3.196 (2)	145 (2)

**Fig. 1.**
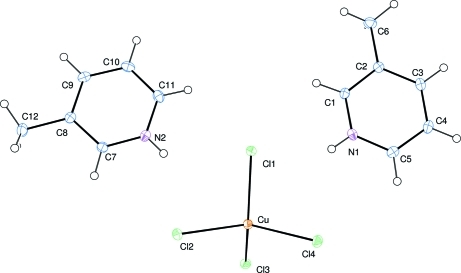
The molecular structure of the title compound, showing displacement ellipsoids drawn at the 30% probability level.

**Fig. 2.**
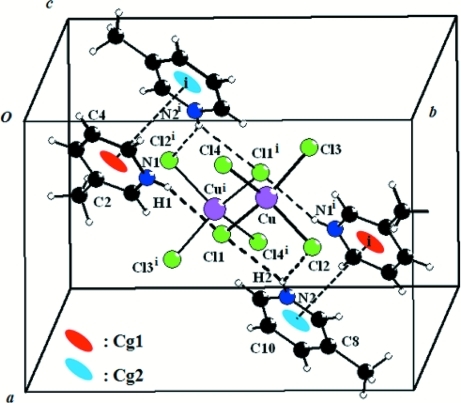
N—H···Cl and π—π interactions (dotted lines) in the title compound. Cg denotes the ring centroid. [Symmetry code: (i) -x+1, -y+1, -z+1]

## supplementary crystallographic information

### Comment

The four–coordinated tetrahalocuprate (II) ions, [CuCl_4_]^2-^ possess a variety of geometries from square planar to near tetrahedral symmetry, and the geometry of tetrahalocuprate (II) species is influenced by the crystal-packing forces resulted from the size and shape of counter cations (Schneider *et al.*, 2007; Parent *et al.*, 2007), hydrogen bonding to cations (Haddad *et al.*, 2006; Marzotto *et al.*, 2001; Choi *et al.*, 2002), and halide-halide interactions in solid (Awwadi *et al.*, 2007). Herein, we report the crystal structure of the title compound, bis(3-methylpyridinium)tetrachloridocuprate(II) (Fig. 1).

The [CuCl_4_]^2-^ anion in the title compound is distorted to be approximately D_2d_, somewhat distorted from tetrahedral as a result of hydrogen bonding interactions with two 3-methylpyridinium cations. The range of Cl—Cu—Cl angles is 99.28 (2)–137.32 (2)°, which is far away from tetrahedral geometry. The Cl1 atom of [CuCl_4_]^2-^ anion forms a three-center hydrogen bond with two protonated pyridinium N atoms (Fig. 2 & Table 1). On the other hand, the Cl2 atom forms a common two–center hydrogen bond with nicotinium cations. As shown in Fig. 2, there are weak π—π interactions between pyridinium rings of two discrete units. The Cg1···Cg2^i^ distance is 3.704 (2) Å (Cg1 and Cg2 are the centroids of the C1–C5/N1 pyridinium ring and the C7–C11/N2 pyridinium ring, respectively; symmetry code as in Fig.2 & Table 1).

### Experimental

A total 1 mmol (0.093 g) of 3-methylpyridine and 1 mmol (0.170 g) of CuCl_2_.2H_2_O were dissolved in 10 mL of ethanol acidified with 5 mL of concentrated HCl. The mixture solution was heated and refluxed for 1 hr. The single crystals were obtained by slow evaporation in ethanol solution for 7 days. Elemental analysis was performed at the Korean Basic Science Center. Anal. (%) calculated for C_12_H_16_Cl_4_CuN_2_: C, 36.62; H, 4.10; N, 7.12; found: C, 37.23; H, 4.33; N, 7.17. Spectroscopic analysis: The electronic spectrum in DMF solution: ν_1_; 874 nm (ε = 20*M*^-1^cm^-1^), ν_2_; 1038 (71), ν_3_; 1326 (62). The peak was analyzed into three peaks  based on the distorted tetrahedral structure around copper(II) metal ion (Lee *et al.*, 2002). These bands are tentatively assigned to d*_x_*^2^_-_*_y_*^2^(^2^B_2_) —> d*_xz_*, d*_yz_*(^2^E), d*_x_*^2^_-_*_y_*^2^ —> d*_xy_*(^2^B_1_), d*_x_*^2^_-_*_y_*^2^ —> d*_z_*^2^(^2^A_1_), respectively.

### Refinement

The H1 and H8 atoms were located in a difference map and refined freely. Other H atoms were positioned geometrically and refined using a riding model, with C—H = 0.93 - 0.96 Å, and with *U*_iso_(H) = 1.2*U*_eq_(C) for aromatic and 1.5*U*_eq_(C) for methyl H atoms.

### Crystal data

**Table:**

(C_6_H_8_N)_2_[CuCl_4_]	*F*(000) = 796
*M**_r_* = 393.61	*D*_x_ = 1.658 Mg m^−^^3^
Monoclinic, *P*2_1_/*n*	Mo *K*α radiation, λ = 0.71073 Å
Hall symbol: -P 2yn	Cell parameters from 7316 reflections
*a* = 9.0438 (3) Å	θ = 2.8–28.3°
*b* = 13.0530 (4) Å	µ = 2.05 mm^−^^1^
*c* = 13.7391 (5) Å	*T* = 123 K
β = 103.541 (2)°	Block, orange
*V* = 1576.80 (9) Å^3^	0.25 × 0.24 × 0.23 mm
*Z* = 4	

### Data collection

**Table:**

Bruker SMART CCD area-detector diffractometer	3429 reflections with *I* > 2σ(*I*)
φ and ω scans	*R*_int_ = 0.036
Absorption correction: multi-scan (*SADABS*; Bruker, 2002)	θ_max_ = 28.3°, θ_min_ = 2.2°
*T*_min_ = 0.603, *T*_max_ = 0.62	*h* = −11→12
16009 measured reflections	*k* = −17→17
3899 independent reflections	*l* = −17→18

### Refinement

**Table:**

Refinement on *F*^2^	0 restraints
Least-squares matrix: full	H atoms treated by a mixture of independent and constrained refinement
*R*[*F*^2^ > 2σ(*F*^2^)] = 0.024	*w* = 1/[σ^2^(*F*_o_^2^) + (0.0228*P*)^2^ + 0.7012*P*] where *P* = (*F*_o_^2^ + 2*F*_c_^2^)/3
*wR*(*F*^2^) = 0.062	(Δ/σ)_max_ = 0.001
*S* = 1.04	Δρ_max_ = 0.36 e Å^−^^3^
3899 reflections	Δρ_min_ = −0.35 e Å^−^^3^
182 parameters	

### Special details

**Table:**

Geometry. All esds (except the esd in the dihedral angle between two l.s. planes) are estimated using the full covariance matrix. The cell esds are taken into account individually in the estimation of esds in distances, angles and torsion angles; correlations between esds in cell parameters are only used when they are defined by crystal symmetry. An approximate (isotropic) treatment of cell esds is used for estimating esds involving l.s. planes.

### Fractional atomic coordinates and isotropic or equivalent isotropic displacement parameters (Å^2^)

**Table:**

	*x*	*y*	*z*	*U*_iso_*/*U*_eq_	
Cu	0.55797 (2)	0.545725 (15)	0.703075 (14)	0.01588 (6)	
Cl1	0.65531 (4)	0.43858 (3)	0.60513 (3)	0.02068 (9)	
Cl2	0.75323 (4)	0.65743 (3)	0.73115 (3)	0.02099 (9)	
Cl3	0.36357 (4)	0.65699 (3)	0.68081 (3)	0.01929 (9)	
Cl4	0.46771 (5)	0.42349 (3)	0.78922 (3)	0.02114 (9)	
N1	0.44305 (15)	0.24760 (11)	0.59904 (10)	0.0181 (3)	
H1	0.486 (2)	0.2980 (17)	0.6238 (15)	0.031 (6)*	
C1	0.46256 (17)	0.21958 (12)	0.50880 (11)	0.0172 (3)	
H1A	0.5226	0.2594	0.4771	0.021*	
C2	0.39398 (17)	0.13197 (12)	0.46271 (11)	0.0175 (3)	
C3	0.30426 (18)	0.07611 (13)	0.51325 (12)	0.0207 (3)	
H3	0.2563	0.0168	0.4843	0.025*	
C4	0.28506 (18)	0.10739 (13)	0.60617 (12)	0.0214 (3)	
H4	0.2243	0.0698	0.6392	0.026*	
C5	0.35733 (18)	0.19485 (13)	0.64851 (12)	0.0198 (3)	
H5	0.3467	0.217	0.7108	0.024*	
C6	0.4135 (2)	0.10069 (14)	0.36131 (12)	0.0244 (4)	
H6A	0.3316	0.1282	0.3106	0.037*	
H6B	0.413	0.0273	0.3567	0.037*	
H6C	0.5086	0.1266	0.3519	0.037*	
N2	0.85716 (15)	0.61542 (11)	0.52789 (10)	0.0188 (3)	
H2	0.820 (3)	0.5984 (19)	0.5735 (17)	0.044 (7)*	
C7	0.94635 (17)	0.69916 (13)	0.54089 (11)	0.0182 (3)	
H7	0.9581	0.7377	0.5992	0.022*	
C8	1.02028 (17)	0.72809 (12)	0.46826 (11)	0.0178 (3)	
C9	0.99762 (18)	0.66842 (13)	0.38198 (12)	0.0197 (3)	
H9	1.0457	0.6861	0.3315	0.024*	
C10	0.90430 (19)	0.58299 (13)	0.37064 (12)	0.0223 (3)	
H10	0.8895	0.5436	0.3128	0.027*	
C11	0.83398 (19)	0.55690 (13)	0.44509 (12)	0.0214 (3)	
H11	0.7712	0.4997	0.4386	0.026*	
C12	1.1178 (2)	0.82208 (13)	0.48099 (13)	0.0253 (4)	
H12A	1.0547	0.8815	0.4636	0.038*	
H12B	1.1875	0.8177	0.4381	0.038*	
H12C	1.1738	0.8273	0.5494	0.038*	

### Atomic displacement parameters (Å^2^)

**Table:**

	*U*^11^	*U*^22^	*U*^33^	*U*^12^	*U*^13^	*U*^23^
Cu	0.01598 (11)	0.01411 (10)	0.01823 (11)	−0.00095 (7)	0.00540 (8)	−0.00009 (7)
Cl1	0.0225 (2)	0.01704 (18)	0.0254 (2)	−0.00297 (15)	0.01148 (16)	−0.00423 (14)
Cl2	0.02040 (19)	0.02015 (19)	0.02291 (19)	−0.00627 (15)	0.00604 (15)	−0.00435 (15)
Cl3	0.02013 (19)	0.01763 (18)	0.02150 (19)	0.00232 (14)	0.00768 (15)	0.00053 (14)
Cl4	0.0242 (2)	0.01839 (19)	0.02248 (19)	−0.00029 (15)	0.00882 (15)	0.00461 (14)
N1	0.0172 (6)	0.0156 (7)	0.0202 (7)	−0.0019 (5)	0.0016 (5)	−0.0005 (5)
C1	0.0163 (7)	0.0172 (7)	0.0186 (7)	0.0000 (6)	0.0048 (6)	0.0028 (6)
C2	0.0168 (7)	0.0177 (7)	0.0174 (7)	0.0020 (6)	0.0028 (6)	0.0017 (6)
C3	0.0197 (8)	0.0171 (8)	0.0243 (8)	−0.0027 (6)	0.0029 (6)	0.0012 (6)
C4	0.0189 (8)	0.0215 (8)	0.0245 (8)	−0.0009 (6)	0.0066 (6)	0.0061 (7)
C5	0.0184 (7)	0.0233 (8)	0.0177 (7)	0.0030 (6)	0.0042 (6)	0.0025 (6)
C6	0.0300 (9)	0.0238 (9)	0.0192 (8)	0.0011 (7)	0.0053 (7)	−0.0024 (7)
N2	0.0180 (7)	0.0204 (7)	0.0185 (7)	−0.0002 (5)	0.0056 (5)	0.0027 (5)
C7	0.0186 (8)	0.0183 (8)	0.0173 (7)	0.0003 (6)	0.0034 (6)	−0.0004 (6)
C8	0.0157 (7)	0.0185 (8)	0.0191 (7)	0.0030 (6)	0.0039 (6)	0.0029 (6)
C9	0.0206 (8)	0.0228 (8)	0.0162 (7)	0.0069 (6)	0.0056 (6)	0.0042 (6)
C10	0.0254 (8)	0.0225 (8)	0.0172 (7)	0.0039 (7)	0.0015 (6)	−0.0035 (6)
C11	0.0196 (8)	0.0186 (8)	0.0238 (8)	−0.0006 (6)	0.0005 (6)	0.0001 (6)
C12	0.0263 (9)	0.0237 (9)	0.0272 (9)	−0.0058 (7)	0.0089 (7)	0.0020 (7)

### Geometric parameters (Å, °)

**Table:**

Cu—Cl3	2.2455 (4)	C6—H6B	0.96
Cu—Cl4	2.2506 (4)	C6—H6C	0.96
Cu—Cl2	2.2526 (4)	N2—C7	1.345 (2)
Cu—Cl1	2.2576 (4)	N2—C11	1.345 (2)
N1—C5	1.336 (2)	N2—H2	0.81 (2)
N1—C1	1.343 (2)	C7—C8	1.378 (2)
N1—H1	0.80 (2)	C7—H7	0.93
C1—C2	1.382 (2)	C8—C9	1.393 (2)
C1—H1A	0.93	C8—C12	1.497 (2)
C2—C3	1.391 (2)	C9—C10	1.385 (2)
C2—C6	1.501 (2)	C9—H9	0.93
C3—C4	1.389 (2)	C10—C11	1.368 (2)
C3—H3	0.93	C10—H10	0.93
C4—C5	1.375 (2)	C11—H11	0.93
C4—H4	0.93	C12—H12A	0.96
C5—H5	0.93	C12—H12B	0.96
C6—H6A	0.96	C12—H12C	0.96
			
Cl3—Cu—Cl4	99.283 (16)	C2—C6—H6C	109.5
Cl3—Cu—Cl2	99.327 (17)	H6A—C6—H6C	109.5
Cl4—Cu—Cl2	137.322 (16)	H6B—C6—H6C	109.5
Cl3—Cu—Cl1	136.189 (16)	C7—N2—C11	122.92 (15)
Cl4—Cu—Cl1	96.568 (17)	C7—N2—H2	117.7 (17)
Cl2—Cu—Cl1	95.946 (16)	C11—N2—H2	119.3 (17)
C5—N1—C1	123.14 (15)	N2—C7—C8	120.27 (15)
C5—N1—H1	119.2 (15)	N2—C7—H7	119.9
C1—N1—H1	117.7 (15)	C8—C7—H7	119.9
N1—C1—C2	120.43 (15)	C7—C8—C9	117.62 (15)
N1—C1—H1A	119.8	C7—C8—C12	120.84 (15)
C2—C1—H1A	119.8	C9—C8—C12	121.53 (15)
C1—C2—C3	117.17 (15)	C10—C9—C8	120.68 (15)
C1—C2—C6	120.83 (15)	C10—C9—H9	119.7
C3—C2—C6	121.98 (15)	C8—C9—H9	119.7
C4—C3—C2	121.16 (15)	C11—C10—C9	119.60 (15)
C4—C3—H3	119.4	C11—C10—H10	120.2
C2—C3—H3	119.4	C9—C10—H10	120.2
C5—C4—C3	118.91 (16)	N2—C11—C10	118.90 (16)
C5—C4—H4	120.5	N2—C11—H11	120.5
C3—C4—H4	120.5	C10—C11—H11	120.5
N1—C5—C4	119.19 (16)	C8—C12—H12A	109.5
N1—C5—H5	120.4	C8—C12—H12B	109.5
C4—C5—H5	120.4	H12A—C12—H12B	109.5
C2—C6—H6A	109.5	C8—C12—H12C	109.5
C2—C6—H6B	109.5	H12A—C12—H12C	109.5
H6A—C6—H6B	109.5	H12B—C12—H12C	109.5

### Hydrogen-bond geometry (Å, °)

**Table:**

*D*—H···*A*	*D*—H	H···*A*	*D*···*A*	*D*—H···*A*
N1—H1···Cl1	0.80 (2)	2.44 (2)	3.136 (2)	146 (2)
N2—H2···Cl1	0.81 (2)	2.66 (2)	3.270 (2)	134 (2)
N2—H2···Cl2	0.81 (2)	2.50 (2)	3.196 (2)	145 (2)
